# Effect of Delayed Icing on Biogenic Amines Formation and Bacterial Contribution of Iced Common Carp (*Cyprinus carpio*)

**DOI:** 10.3390/molecules181215464

**Published:** 2013-12-12

**Authors:** Seyed Vali Hosseini, Ali Hamzeh, Mehran Moslemi, Aria Babakhani Lashkan, Antonio Iglesias, Xesús Feás

**Affiliations:** 1 Department of Fisheries, University of Tehran, Karaj 31587-77871, Iran; E-Mail: hosseinisv@ut.ac.ir; 2 Department of Seafood Science and Technology, Tarbiat Modares University, Noor 64414-356, Iran; E-Mail: ahamze86@gmail.com; 3 Department of Fisheries, Islamic Azad University, Jouybar Branch, Jouybar 47715-195, Iran; E-Mail: m_moslemi1000@yahoo.com; 4 Department of Fisheries, University of Guilan, Sowmeh Sara 1144, Iran; E-Mail: arialashkan@gmail.com; 5 Department of Anatomy and Animal Production, Faculty of Veterinary, University of Santiago de Compostela, Lugo E-27002, Galicia, Spain; E-Mail: antonio.iglesias@usc.es; 6 Department of Organic Chemistry, Science Faculty, University of Santiago de Compostela, Lugo E-27002, Galicia, Spain

**Keywords:** delayed icing, biogenic amines, storage, fish, carp

## Abstract

The variation of six biogenic amines (BAs) and total viable count (TVC) in common carp (*Cyprinus carpio*) stored in ice with 0, 4 and 8 h delay before icing was evaluated in a period of 4 days. Delayed icing led to significant (*p* < 0.05) increases in TVC throughout the period of storage and showed a good correlation with BAs content. The obtained data showed that putrescine and cadaverine were predominant in all samples and it was indicated that they could be proper indicators to determine the carp quality. Spermidine and spermine increased slightly toward the end of storage and the levels of dangerous BAs (histamine and tyramine) were under the limit over the period. As a result, it is indicated that delaying time affects on formation of BAs and the effect in samples with 8 h delay was significantly (*p* < 0.05) more than those with 0 and 4 h delay.

## 1. Introduction

Fresh fish quality is influenced by many factors, including the temperature [1]. One of the oldest and most effective methods of fish preservation is icing and ice storage. Delay in icing or improper handling of fish after catch may result in quality degradation [2]. The decomposition of fish proteins yields peptides and amino acids, which are susceptible to further degradation, resulting in the formation of biogenic amines (BAs), which can be widely distributed in proteinaceous foods [3]. 

BAs such as putrescine (PUT), cadaverine (CAD), spermidine (SPD), spermine (SPM), histamine (HIS) and tyramine (TYR) are non-volatile and low-molecular weight compounds and are generally produced by microbial decarboxylation of specific amino acids [4,5,6]. The amount and type of BAs formed is related to the initial quality of the raw material, the availability of free amino acids, the presence of bacterial biogenic amine decarboxylases and the temperature [7,8]. Determination of BAs in proteinaceous foodstuff is important because they can impact human health and can be used as quality indicators. BAs play an important role in the degradation pathway of amino acids and since they are produced by spoilage bacteria toward the end of shelf-life, their level can represent the quality of food [6,9].

BAs are biosynthesized at very low levels in fresh fish and are necessary to good physiological function such as growth regulation (SPM, SPD, CAD), neural transition (catecholamine and serotonin) and mediators of inflammation (HIS and TYR) [10], but in larger quantities the can cause health problems in humans [7]. 

The toxicity of amines depends strongly on the individual detoxification efficiency [11]. During the digestion process in the human gut, low amounts of biogenic amines are metabolized to physiologically less-active degradation products. This detoxification system includes specific enzymes (e.g., diamine oxidase, DAO). In fact, under normal condition, BAs can be detoxified by amine oxidase or by conjugation, but upon intake of high loads of BAs with foods or in sensitive people this detoxification system is unable to sufficiently eliminate BAs or these procedures may be disrupted by the presence of inhibitory agents of the enzyme, so afterwards they can be accumulated and cause poisoning [8,12]. Moreover, in the case of insufficient DAO activity, caused by e.g., genetic predisposition, gastrointestinal diseases or inhibition of DAO activity due to secondary effects of medicines or alcohol, even low amounts of biogenic amines cannot be metabolized efficiently [13]. 

BAs are structurally classified to aliphatic (PUT, CAD, SPM, SPD), aromatic (TYR and phenylethylamine) and heterocyclic (HIS and tryptamine) [14,15]. Among them, histamine is more dangerous and its toxicity can be enhanced by the presence of other amines such as PUT and CAD. Moreover, biogenic polyamines (PUT, CAD, SPM, and SPD) are potential carcinogenic substances that can be converted into nitrosamines when exposed to nitrite [12,16].

Although in many researches, the BA content of marine fish has been determined, there is little information about these components in freshwater fish [7,17]. On the other hand, common carp (*Cyprinus carpio*) is an important freshwater fish in global aquaculture, and is by far the most important freshwater fish in Iran as well. In Iran, and many other countries, this fish is mainly offered as whole (guts in) fish on ice for sale. In some cases, fish may spend several hours at ambient temperature, before icing or without ice. High ambient temperatures and long pre-icing periods may accelerate the deterioration of fish quality. Thus, the objective of the present study was to determine how icing delays can affect BA formation and bacterial populations in common carp muscle.

## 2. Results and Discussion

### 2.1. Biogenic Amines

The concentration of BAs presented in common carp stored in ice with 0, 4 and 8 h delay are shown in Table 1. PUT and CAD were the predominant amines in all samples and their levels were clearly higher than those of the other amines. Therefore they can be a good quality marker to show the quality of common carp specimens. Křížek *et al.*, have suggested that PUT and CAD and the sum of both amines are useful quality indicators for common carp flesh [7], whereas Dawood *et al.*, also stated that these two amines can be used to assess freshness of chill-stored rainbow trout [18]. In addition, Křížek *et al.*, suggested that PUT values lower than 10 mg kg^−1^ can represent the good quality of the common carp flesh, 10–20 mg kg^−1^ as acceptable quality and the value over than 20 can indicate the poor quality established on sensory evaluation [19]. 

**Table 1:** Changes in biogenic amines contents (mg kg^−1^) of common carp stored in ice with 0, 4 and 8 h delay before icing.

				Day		
BAs	Delay	0	4	8	12	16
PUT	0	1.3 ± 0.47^ bE^	4.19 ± 0.68 ^bD^	13.65 ± 1.23 ^bC^	30.71 ± 2.13 ^bB^	91.93 ± 1.98 ^bA^
	4	1.53 ± 0.54^ bD^	6.29 ± 0.47 ^bD^	20.11 ± 2.65 ^bC^	41.33 ± 2.49 ^bB^	88.43 ± 8.05^ bA^
	8	11.43 ± 2.1^ aC^	16.61 ± 3.03 ^aC^	60.73 ± 6.86 ^aB^	174.57 ± 20.03^aA^	171 ± 7.21 ^aA^
CAD	0	ND	6.71 ± 1.38 ^bD^	22.49 ± 3.95 ^cC^	52.87 ± 3.2 ^bB^	128.4 ± 11.96 ^bA^
	4	3.71 ± 1.4 ^bC^	10.51 ± 2.62^ bC^	51.06 ± 6.24 ^bB^	42.27 ± 1.97 ^bB^	123.47 ± 12.07^bA^
	8	16.05 ± 3.53 ^aC^	59.73 ± 9.72^ aB^	159.27 ± 6.72^ aA^	150.87 ± 4.92^ aA^	158.53 ± 16.92^Aa^
SPM	0	7.66 ± 0.57 ^bD^	8.79 ± 0.71 ^bC^	8.52 ± 0.23 ^bC^	10.66 ± 0.86 ^cB^	12.73 ± 0.5 ^aA^
	4	7.65 ± 1.55 ^bB^	8.82 ± 0.61 ^bB^	8.86 ± 0.24 ^bB^	12.03 ± 0.58 ^bA^	13.2 ± 0.4 ^aA^
	8	10.93 ± 0.71^aC^	11.7 ± 1.25^aBC^	12.4 ± 0.96 ^aAB^	13.63 ± 0.28 ^aA^	12.41 ± 0.2 ^aAB^
SPD	0	6.86 ± 0.71 ^aC^	8.07 ± 0.36 ^aB^	6.1 ± 0.37 ^bC^	8.62 ± 0.39 ^bB^	10.04 ± 1.06 ^bA^
	4	6.19 ± 0.72 ^aB^	8.63 ± 0.53 ^aB^	11.97 ± 1.11^aA^	12.08 ± 1.7 ^aA^	11.4 ± 1.21 ^abA^
	8	7.45 ± 0.88 ^aB^	8.48 ± 0.71 ^aB^	12.73 ± 3.14^aA^	11.9 ± 1.05 ^aA^	12.66 ± 0.5 ^aA^
HIS	0	ND	ND	0.23 ± 0.06 ^cB^	0.32 ± 0.04 ^bB^	0.47 ± 0.04 ^bA^
	4	ND	0.24 ± 0.07^ bB^	0.36 ± 0.05 ^bB^	0.31 ± 0.1 ^bB^	0.531 ± 0.06^ bA^
	8	0.14 ± 0.04^ C^	0.43 ± 0.1 ^aB^	0.6 ± 0.01 ^aA^	0.49 ± 0.01 ^aB^	0.67 ± 0.05 ^aA^
TYR	0	ND	ND	ND	0.45 ± 0.07 ^cB^	1.12 ± 0.2 ^bA^
	4	ND	ND	0.61 ± 0.16 ^aB^	1.62 ± 0.31 ^bA^	2.04 ± 0.38^ aA^
	8	0.28 ± 0.042 ^C^	0.72 ± 0.11 ^C^	1.41 ± 0.43 ^aB^	2.35 ± 0.18 ^aA^	2.38 ± 0.55 ^aA^

^a, b, c^ Means with different superscript letters in the same column represent significant difference in different delay time at *p < 0.05*; ^A, B, C, D, E^ Means with different superscript letters in the same row represent significant difference during the storage time at *p < 0.05*; BAs: Biogenic Amines. PUT: Putrescine, CAD: Cadedverine, SPM: Spermine, HIS: Histamine, SPD: Spermidine, TYR: Tyramine; ND: Not Detected.

In the present study the PUT value reached over 20 mg kg^−1^ on day 12 for samples without delayed icing, while it was more than 20 mg kg^−1^ after 8 days for the samples with 4 and 8 h delays. The maximum value for PUT was observed on day 12 for 8 h delay (174.6 mg kg^−1^) and the value of sample with 8 h delay was higher than for 0 and 4 h delay (*p* < 0.05). CAD was not detected on the first day of the study in samples without delayed icing, but towards the end of storage the values of samples with 0 and 4 h delay was similar and also were lower than samples with 8 h delay (*p* < 0.05).

**Figure 1:**
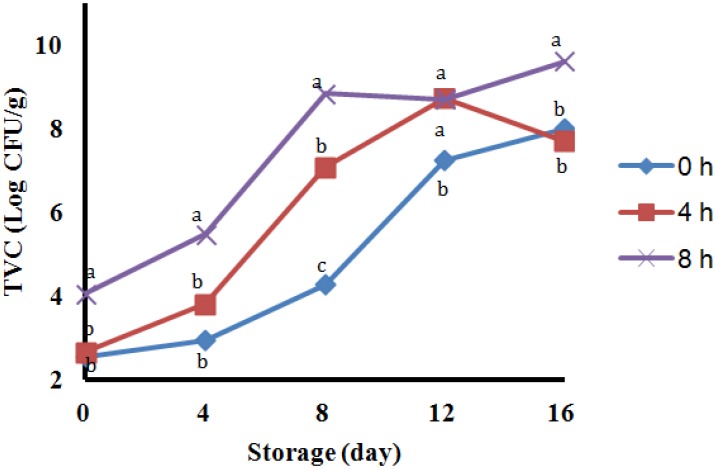
Changes in total viable counts (TVC) of carp stored in ice with 0, 4 and 8 h delay before icing.

The higher levels of PUT and CAD in samples with 8 h delay may be related to higher counts of total volatile counts (TVC) in the samples (Figure 1). The present data are in agreement with those in [7] and [19], who reported PUT and CAD values in common carp flesh were higher than those of other amines and suggested that these two amines can be a good marker to estimate the quality of common carp flesh.

SPD and SPM naturally exist in food, and are not toxicologically important and their contents cannot be a good indicator to determinate carp flesh quality [7,20]. In this study SPD and SPM levels increased slightly during the storage time and samples with 8 h delay had higher amounts of them than the two other samples. The data was in agreement with Ababouch *et al.*, [21], who reported that the formation of SPD and SPM in sardines was inhibited by icing and at ambient temperature their levels reached 6 and 5 mg 100 g^−1^ after 24 h, respectively. Özogul *et al.*, observed that the SPD and SPM in herring did not increase during the storage period and proposed that the SPD and SPM formation are not related to bacterial spoilage [22]. 

For TYR there is no suggested allowable level in food, unlike for other amines. Nevertheless, high levels of TYR can be considered a hypertension risk and it can also be converted into a mutagenic compound, 3-diazotyramine (3-DT), upon treatment with nitrite under acidic conditions [23]. In the present study, TYR was not detectable in the samples with 0 and 4 h delay until day 12 and 8, respectively. The maximum level was found in samples with 8 h delay and on the last day (2.38 mg kg^−1^) and generally the samples with 4 and 8 h delay had higher levels than samples without delay (*p* < 0.05). The results were in agreement with Paoleologos *et al.*, [24]. They reported that TYR was not detected in the early stages of whole, gutted and filleted Mediterranean Sea bass stored on ice and the first appearance was on days 9, 9 and 7 respectively.

HIS was not found in the early stages of storage in samples with 0 and 4 h delay. All obtained data were under the limit and there was no risks of HIS in common carp with different conditions over the storage time. Although all data was under the risk limit, the samples with 8 h delay had significantly higher concentrations of HIS than the two other samples (*p* < 0.05). Mendes reported that the production of HIS in the muscle of sardine and Atlantic horse mackerel stored in ice was negligible and not affected by bacterial growth [25]. Křížek *et al.*, found that HIS content in common carp roe was very low, even in samples with negative sensory characteristics [3]. Křížek *et al.*, reported that the HIS content in vacuum and non-vacuum packaged carp stored at 3 °C was poor [7]. Our data were in agreement with [7] and [9]. Furthermore, BAs, such as HIS and TYR, are considered as antinutritional compounds. For sensitive individuals they represent a health risk, especially when their effect is potentiated by other substances [7]. Poisoning by histamine with its allergy-like symptoms is usually related to the consumption of scombroid fish, such as tuna or mackerel [26], and it is considered to be one of the most common reported forms of food intoxication.

### 2.2. Quality Index (QI) and Biogenic Amine Index (BAI)

As previously mentioned, the importance of BAs is related to their toxicological effects and their advantages for the assessment of the quality of fish and fish products. In this regard, a quality index (QI) was proposed by Mietz and Karmas [27] based on the content of the HIS, CAD, PUT, SPM, and SPD to evaluate the quality of rockfish, tuna, salmon, lobster and shrimp, and in addition biogenic amines index (BAI) was suggested by Veciana-Nogues *et al.*, [28]. The QI and BAI of samples stored in ice with 0, 4 and 8 h delay are shown in Table 2. In this study, QI and BAI was significantly increased (*p <* 0.05) by holding fish samples at ambient temperature for 4 or 8 h on all days. However, the BAI was generally higher than QI, these two indexes were increased during the storage time, and therefore they can be good indices to determine the quality of common carp stored in ice. The data were in agreement with Özogul *et al.*, who reported that BAI was approximately twice the QI, nevertheless both indices increased during the storage of sardines in air, VP and MAP [29]. Bakar *et al.*, reported that BAI and QI increased during the storage of barramundi slices stored in 0 and 4 °C [14]. The present study is in agreement with [20,23]. Furthermore, Mieltz and Karmas proposed the value of 10 for the QI as the limit of fish acceptability [27]. On the basis of this scale and according to QI values, experimental fish could not be consumed after 8 days, for the 8 h iced group. Since, BAs are produced mainly by bacterial and enzymatic activity of fish flesh, the higher levels of BAI and QI of samples iced after 4 or 8 h throughout the period of storage in ice could account for the higher biochemical activity of common carp. Veciana-Nogues *et al.*, stated that BAI is correlated with storage time. However, more studies on BAI are needed to set a limit of fish acceptability during storage [28].

**Table 2:** Quality and biogenic amine indexes of common carp stored in ice with 0, 4 and 8 h delay before icing.

				Day		
	Delay	0	4	8	12	16
**QI**	**0**	0.083 ± 0.02 ^cE^	0.61 ± 0.05 ^bD^	2.33 ± 0.27 ^cC^	4.15 ± 0.44 ^bB^	9.3 ± 0.3^ bA^
	**4**	0.36 ± 0.03 ^bC^	0.93 ± 0.14 ^bC^	3.29 ± 0.31^ bB^	3.55 ± 0.22^ bB^	8. 3 ± 0.66 ^bA^
	**8**	1.44 ± 0.17 ^aD^	3.62 ± 0.27^aC^	8.47 ± 0.43 ^Ab^	12.32 ± 1.24 ^aA^	12.67 ± 1.01 ^aA^
**BAI**	**0**	1.3 ± 0.47 ^bD^	10.9 ± 1 ^bD^	36.37 ± 3.18 ^cC^	84.35 ± 4.3 ^bB^	221.93 ± 11.23 ^bA^
	**4**	5.31 ± 0.99 ^bD^	17.03 ± 2.35^bD^	72.14 ± 3.61 ^bC^	90.53 ± 3.92 ^bB^	214.37 ± 16.28 ^bA^
	**8**	28.35 ± 5.57 ^aD^	77.49 ± 9.9 ^aC^	222.01 ± 13.07 ^aB^	328.28 ± 18.12 ^aA^	332.59 ± 23.33 ^aA^

^a, b, c^ Means with different superscript letters in the same column represent significant difference in different delay time at *p* < 0.05; ^A, B, C, D, E^ Means with different superscript letters in the same row represent significant difference during the storage time at *p* < 0.05; QI = Quality index = (HIS + PUT + CAD) / (1 + SPM + SPD). BAI = Biogenic amines index = (HIS + PUT + CAD + TYR).

### 2.3. Total Viable Count (TVC)

The TVC level of three different samples is given in Figure 1. Delayed icing lead to significant increases (*p* < 0.05) in TVC throughout the period of storage and showed a good correlation with BA content. The samples with 8 h delay had the higher TVC than the other samples (*p* < 0.05) followed by 4 h and the samples without delay. Initial counts of samples were 2.55, 2.65 and 4.04 log CFU g^−1^ and they reached to 4.27, 7.08 and 8.83 log CFU g^−1^ in 8 days with 0, 4 and 8 h delay, respectively. Bakar *et al.*, reported the 6 log CFU g^−1^ is supposed to be the limit of acceptability, therefore the TVC content reached over the maximum in the day 12, 8 and 8, and the shelf-life was nearly 12, 8 and 8 days in samples with 0, 4 and 8 h delaying, respectively [20]. In the present study the TVC levels had to do with PUT level and are in agreement with [20] as they reported that TVC in barramundi stored in ice was lower than samples stored in 4 °C.

## 3. Experimental

### 3.1. Sample Preparation

Forty five specimens of common carp representing the size range commercially available to customers (mean weight = 670 ± 25 g) were collected randomly from a local fish farm (Noor City, Mazandaran Province, Iran) in June 2013. Immediately after collection, fish were washed with distilled water and then divided into three lots (15 fish in each lot). One lot was immediately iced after catch, and the remaining two lots after holding at ambient temperature (24–26 °C) for 4 and 8 h, were iced in styropor boxes (2 ± 1 °C) with outlets for water drainage. The fish to ice ratio was approximately 1:3 and the thickness of the ice layer was ~5 cm. During the experiments some ice was added to the boxes to replace melted ice. Experimental fish were kept at ice for 16 days and at each sampling time (0, 4, 8, 12 and 16 days), three randomly chosen fish were removed from ice and their microbial and biogenic amine contents was determined. 

### 3.2. Total Viable Count (TVC)

At each storage interval, the skin from the anterior dorsal area of each sample was first cleaned with alcohol and then aseptically removed using sterilized scalpels. Then 10 g of flesh with both white and dark muscle was taken and transferred into 90 mL 0.1% peptone water (0118-17-0, Difco, Detroit, MI, USA), homogenized using a Stomacher Lab-Blender (Seward type 400, London, UK) for 1 min. From this dilution, other serial decimal dilutions were prepared. In this study, TVC were determined using plate count agar (Oxide Inc., London, UK), according to the standard American Public Health Association method by counting the colony forming units (log_10_ CFU g^−1^) after incubating the plates at 30 °C for 48 h. Microbial analyses were performed in triplicate on three subsamples of each of the replicates [30]. 

### 3.3. Determination of Biogenic Amine (BA) Content

All standard BAs and LC grade materials *i.e.*, methanol, chloroform, butanol, diethyl ether, and *n*-heptane were obtained from Merck Co. (Hamburg, Germany); double distilled and deionized Millipure water (Millipore, Mississauga, ON, Canada) was used for dilution and chromatographic separation. A Waters 1525 HPLC system (Waters Co., Milford, MA, USA) equipped with a Waters 2487 UV-detector set at 254 nm was use for the BA analyses. The column was a reversed phase C18 Waters Spherisorb ODS-2 (250 × 4.60 mm; particle diameter, 5 µm) with a Waters Spherisorb pre-column cartridge (10 mm length) packed with the same material. The mobile phase was an isocratic mixture of methanol:water (62:38 by volume) and the flow rate was 1.1 mL min^−1^ at room temperature. Samples from the dorsal half of each fish (without skin and bones) were used for the BA analysis. Each sample (5 g) was homogenized in a Waring blender (Waring, New Hartford, CT, USA) for 1 min. The procedure for extraction, separation, and quantification described by Paleologos *et al.*, [15] was used and explained in our previously work [31]. According to this procedure a solution of BA is obtained after sample treatment with trichloroacetic acid (6% w/v) and centrifugation (6 × 130 g, 20 min, 4 °C; 236 HK, Hermle, Germany). BA were isolated after derivatization with benzoyl chloride and surfactant mediated cloud point extraction before HPLC separation and quantification. Results are reported as µg kg^−1^ fish muscle.

### 3.4. Quality Index (QI) and Biogenic Amines Index (BAI)

Calculating of the quality index (QI) and biogenic amines index (BAI) were done by the methods described by Mieltz and Karmas [27] and Veciana-Nogues *et al.*, [28] respectively, as follows:

QI = (HIS + PUT + CAD)/(1 + SPM + SPD)

BAI = (HIS + PUT + CAD + TYR)

### 3.5. Statistical Analysis

All measurements were performed in triplicate for each lot, and the mean values ± Standard deviation were reported for each case. The one-way ANOVA and The Duncan’s test was used for analysis and mean comparison using SPSS software (Version 16.0, Chicago, IL, USA). Significance of differences was defined as *p* < 0.05.

## 4. Conclusions

The BAs in common carp stored in ice under three different conditions were evaluated and the data showed that there is no important risk of HIS and TYR as the more dangerous amines in food because their levels were under the limit and negligible over the period. However all BAs increased during the storage, the values of PUT and CAD was clearer than the others and it can indicate that these two amines can be good markers to evaluate carp quality. In this study it is indicated that the temperature and the time passed after catch significantly affected on the levels of BAs and generally the levels in samples with 8 h delay before icing was significantly higher than in the two others.
